# Frequency of New Silent MRI Lesions in Myelin Oligodendrocyte Glycoprotein Antibody Disease and Aquaporin-4 Antibody Neuromyelitis Optica Spectrum Disorder

**DOI:** 10.1001/jamanetworkopen.2021.37833

**Published:** 2021-12-08

**Authors:** Valentina Camera, Leah Holm-Mercer, Ali Asgar Hatim Ali, Silvia Messina, Timotej Horvat, Wilhelm Kuker, Maria Isabel Leite, Jacqueline Palace

**Affiliations:** 1 Nuffield Department of Clinical Neurosciences, University of Oxford, Oxford, United Kingdom; 2 Department of Clinical Neurosciences, John Radcliffe Hospital, Oxford University Hospitals NHS Trust, Oxford, United Kingdom; 3 Department of Neuroradiology, Oxford University Hospitals NHS Trust, Oxford, United Kingdom

## Abstract

**Importance:**

In multiple sclerosis, magnetic resonance imaging (MRI) new silent lesions contribute to the diagnostic criteria, have prognostic value, and are used in treatment monitoring; but in aquaporin-4 antibody neuromyelitis optica spectrum disorder (AQP4-NMOSD), they are rare between attacks. Their frequency and their association with relapses in adults with myelin oligodendrocyte glycoprotein antibody disease (MOGAD) are still unclear.

**Objective:**

To examine the frequency and characteristics of MRI new silent lesions in MOGAD and AQP4-NMOSD.

**Design, Setting, and Participants:**

This retrospective cohort study analyzed clinical and MRI data of 404 patients with MOGAD or AQP4-NMOSD between February 1, 1994, and April 1, 2021; data were prospectively recorded on the Oxford NMOSD clinical database under follow-up. The study was conducted at the Oxford National Referral Center for NMOSD. Participants included patients with MOGAD and AQP4-NMOSD who were treated within the Oxford National NMO Specialist Service.

**Exposures:**

Seropositive MOGAD and AQP4-NMOSD patients who had MRIs during attacks and the remission phase of their disease.

**Main Outcomes and Measures:**

Frequency of new silent lesions detected by either attack MRIs (during the acute clinical event) or remission MRIs (performed outside of a relapse and at least 3 months from last attack). Median time to next relapse in the presence of definite (reference MRI performed at least 4 weeks from last attack onset), probable (reference MRI performed during last attack), and no new silent lesions on remission MRIs was also evaluated.

**Results:**

One hundred eighty-two MOGAD patients and 222 AQP4-NMOSD patients were included. Of the MOGAD patients, 113 (62%) were female, median age at onset was 28 years (range, 2-72), and median follow-up was 52 months (range, 11-253). Of the AQP4-NMOSD patients, 189 (85%) were female, median age at onset was 43 years (range, 2-82), and median follow-up was 87.5 months (range, 11-260). MOGAD patients had 296 attack MRI sessions and 167 remission MRI sessions. New attack silent lesions were found in 97 of 296 (33%) attack MRI sessions, whereas new remission silent lesions were found in 5 of 167 (3.0%) remission MRI sessions. Median time from remission scan to the next relapse in the presence of definite or probable new remission lesions was 2 months (IQR, 1-6), whereas in the absence of any new remission lesions it was 73 months (IQR, 20-104; hazard ratio, 23.86; 95% CI, 7.51-75.79; *P* < .001). AQP4-NMOSD patients had 470 attack MRI sessions and 269 remission MRI sessions. New attack silent lesions were detected in 88 of 470 (18.7%) attack MRI sessions, whereas new remission silent lesions were found in 7 of 269 (2.6%) remission MRI sessions. Median time from remission scan to the next relapse in the presence of definite or probable new remission lesions was 5 months (IQR, 2-6), whereas in the absence of any new remission lesions it was 85 months (IQR, 29-167; hazard ratio, 21.23; 95% CI, 8.05-53.65; *P* < .001).

**Conclusions and Relevance:**

In contrast to that reported in multiple sclerosis, results of this cohort study suggest that new remission silent lesions are rare on follow-up scans in MOGAD and AQP4-NMOSD and appear to indicate a high risk of imminent relapse.

## Key Points

**Question:**

How common is silent T2 lesion activity in myelin oligodendrocyte glycoprotein antibody disease (MOGAD) and aquaporin-4 antibody neuromyelitis optica spectrum disorder (AQP4-NMOSD)?

**Findings:**

In this cohort study of 404 patients with MOGAD or AQP4-NMOSD, new remission (silent) T2 lesions occurred in 5 of 167 MOGAD and 7 of 269 AQP4-NMOSD nonrelapse imaging sessions. Median time from magnetic resonance imaging to next relapse was shorter in the presence of new magnetic resonance imaging remission (silent) lesions (2 and 5 months) than in their absence (73 and 85 months) in both MOGAD and AQP4-NMOSD cohorts, respectively.

**Meaning:**

These findings suggest that new silent lesions are rare in MOGAD and AQP4-NMOSD during remission and appear to precede imminent relapses.

## Teaser

This cohort study assesses the frequency and characteristics of new brain and spinal cord silent lesions on magnetic resonance imaging in seropositive patients with myelin oligodendrocyte glycoprotein antibody disease or aquaporin-4 antibody neuromyelitis optica spectrum disorder.

## Introduction

Myelin oligodendrocyte glycoprotein antibody disease (MOGAD) is a relatively newly described demyelinating disease of the central nervous system, without predominance by sex or racial category.^1^ It is characterized by serum MOG IgG1 autoantibodies whose pathogenetic role has not been proven yet. Its clinical phenotype is broad, most frequently presenting with unilateral or bilateral optic neuritis, but also transverse myelitis, brain and brainstem attacks, and a rarer cortical syndrome with seizure.^1,2^ In children, MOGAD accounts for approximately 50% of acute disseminated encephalomyelitis cases.^3^ Approximately 50% of patients have a relapsing course,^2^ although this appears less in children.^3^ Conversely, aquaporin-4 antibody neuromyelitis optica spectrum disorder (AQP4-NMOSD) is a relapsing astrocytopathy affecting preferentially non-White female individuals and requiring long-term immunosuppression to reduce the high rate of relapse-related disability. AQP4-NMOSD may present with severe optic neuritis, longitudinally extensive transverse myelitis, and area postrema, brainstem, diencephalic, and cerebral syndromes.^1,4^ The prompt distinction between MOGAD, AQP4-NMOSD, and multiple sclerosis (MS) may be challenging for clinicians because the diseases share some clinical and magnetic resonance imaging (MRI) characteristics, which may hamper the treatment of patients.

The appearance of new asymptomatic (silent) T2 hyperintense lesions on interval MRI outside of relapses is a characteristic feature used in the diagnosis of MS,^5^ and their absence is one of the treatment goals.^6^ Although MRI silent lesions during attacks have been reported in both the antibody diseases^7,8^ and new brain interattack asymptomatic lesions have been rarely found in AQP4-NMOSD,^9^ the role of new brain and spinal cord silent lesions outside of relapses is unclear in MOGAD, with only a single pediatric study noting they occur in a minority of children with MOGAD.^10^

In this retrospective analysis, we aimed to assess the frequency of new brain and spinal cord silent lesions on clinical MRIs and their association with relapses in a large cohort of patients with MOGAD and AQP4-NMOSD across all ages.

## Methods

### Population

This cohort study retrospectively included patients^11,12,13^ with clinically and serologically diagnosed MOGAD or AQP4-NMOSD who were treated within the Oxford National NMO Specialist Service. Data on race and ethnicity were not collected from all patients because this was not considered a focus of the study, and the collected data were not sufficient to conduct any analysis by race and ethnicity. Participating patients signed a written informed consent to collect their clinical and imaging data according to the Oxford Research Ethics, and the study was approved by the South Central–Oxford C Research Ethics Committee. Data analyzed had been collected from February 1, 1994, to April 1, 2021. This study followed the Strengthening the Reporting of Observational Studies in Epidemiology (STROBE) reporting guideline.

### Clinical Data Collection

We collected demographic and clinical information from the Oxford NMOSD database: sex, date of birth, age at disease onset, age at MOG or AQP4 antibody detection, follow-up duration, total number of clinical demyelinating events (attacks), MRI dates for each patient, date of the last attack before the considered MRI, immunotherapy at the time of the MRI scan, and date of the first clinical relapse after the considered MRI.

### MRI Data Collection and New Lesion Analysis

Magnetic resonance images at 1.5 and 3 T were assessed according to the local respective neuroscience department protocols at the time. All the brain MRI protocols included T2-weighted imaging/fluid-attenuated inverse recovery, T1-weighted imaging, and diffusion-weighted imaging sequences. Whole spinal cord MRIs were acquired, including T2-weighted imaging, T1-weighted imaging, and short tau inversion recovery sequences. Gadolinium contrast was administered variably according to the local protocols and clinical scenario. All the scans were reviewed by the Oxford NMOSD expert neurologists (M.I.L. and J.P.) and neuroradiologists (T.H. and W.K.) and data were recorded on the Oxford NMO database. The MRI scans were classified as attack MRIs if performed during the clinical demyelinating events and as remission MRIs if performed at least 3 months from the last attack and the patient was completely free of new symptoms. The latter were arranged to “rebaseline” the appearances for future comparison and were not performed at regular intervals after the last inflammatory attack. We compared lesions on the MRI to the last reference MRI. On the attack MRIs, we identified new (T2-weighted imaging/fluid-attenuated inverse recovery) attack silent lesions as those unrelated to the clinical symptoms and signs. All brain and brainstem lesions were assumed to be symptomatic when it was not possible to distinguish whether they were symptomatic, such as in acute disseminated encephalomyelitis.

On remission MRIs (all had to have been performed at least 3 months from the last attack and all patients had to be completely free of new symptoms), we identified new (T2-weighted imaging/fluid-attenuated inverse recovery) remission lesions (ie, remission silent lesions), and they were classified as probable if compared with a reference attack MRI and as definite if compared with a reference MRI performed at least 4 weeks after last attack onset. The “definite” category was added to avoid the inclusion of new lesions evolving during the acute attack but after the attack MRI.

Some imaging sessions scanned both brain and spinal cord and some either brain or spinal cord regions according to the anatomic areas affected during the previous relapses. Finally, we recorded time from remission MRI to the next relapse.

### Statistical Analysis

We described continuous variables by using medians and IQRs or ranges and categorical variables by using proportions and percentages. Proportions were compared with the χ^2^ test. Kaplan-Meier curves were used for depicting time from remission MRIs to next relapse, and group comparison was evaluated by log-rank test and Cox proportional hazard ratio. We assessed the Cox proportional hazard assumptions with Schoenfeld residuals. The analysis was conducted with Stata version 14 (StataCorp LLC) in June 2021. Significance was determined at 2-sided *P* = .05.

## Results

### Demographic and Clinical Baseline Data

We analyzed a cohort of 404 patients (302 [75%] female patients; 102 [25%] male patients), 182 with MOGAD with a median follow-up of 52 months (range, 11-253 months) and 222 with AQP4-NMOSD with a median follow-up of 87.5 months (range, 11-260 months). Of the 182 MOGAD patients, 113 (62%) were female, 69 (38%) were male, median age at onset was 28 years (range, 2-75 years), and 49 (27%) had pediatric-onset disease (<18 years at onset). Median time from onset to MOG IgG1 detection was 1 month (range, 0-452 months), and median age at MOG IgG1 detection was 32 years (range, 3-75 years).

Of the 222 AQP4-NMOSD patients, 189 (85%) were female, 33 (15%) were male, median age at onset was 43 years (range, 3-84 years), and 28 (12.6%) had pediatric-onset disease. Median time from onset to AQP4 IgG detection was 4 months (range, 0-314 months), and median age at AQP4 IgG detection was 47.5 years (range, 3-82 years) (Table 1).

**Table 1.** Cohort Characteristics

Characteristic	No. (%)	
	MOGAD (n = 182)	AQP4-NMOSD (n = 222)
Sex		
Female	113 (62.1)	189 (85.1)
Male	69 (37.9)	33 (14.9)
Age, median (range), y		
Current	38 (7-80)	55 (7-90)
At onset	28 (2-75)	43 (3-84)
Age <18 y at disease onset	49 (26.9)	28 (12.6)
No. of attacks per patient, median (range)	2 (1-15)	2.5 (1-23)
Follow-up duration, median (range), mo	52 (11-253)	87.5 (11-260)
**MRI**		
No. of attacks		
Total (sessions)^a^	416 (296)	669 (470)
Brain	265	338
Spinal cord	151	331
No. of remissions		
Total (sessions)^a^	247 (167)	379 (269)
Brain	137	179
Spinal cord	110	200
Age at remission MRI scanning, median (range), y	33 (3-76)	49 (9-86)
Age <18 y at remission MRI^b^	36 (21.5)	21 (7.8)
Therapy at remission MRI scan^b^		
Azathioprine ± prednisolone	15 (9)	104 (38.7)
Mycophenolate ± prednisolone	11 (6.6)	61 (22.7)
Methotrexate ± prednisolone	5 (3)	29 (10.8)
Cyclophosphamide ± prednisolone	0	2 (0.74)
Rituximab ± prednisolone	3 (1.8)	13 (4.8)
Tacrolimus	2 (1.2)	0
IVIG ± prednisolone	0	4 (1.5)
Eculizumab	1 (0.6)	0
Oral prednisolone	51 (30.5)	29 (10.8)
MS therapy	1 (0.6)	1 (0.37)
No/subtherapeutic therapy^c^	78 (46.7)	26 (9.6)

Abbreviations: AQP4-NMOSD, aquaporin-4 antibody neuromyelitis optica spectrum disorder; IVIG, intravenous immunoglobulin; MOGAD, myelin oligodendrocyte glycoprotein antibody disease; MRI, magnetic resonance imaging; MS, multiple sclerosis.

^a^ Brain and spinal cord scans often performed during a single MRI session.

^b^ Denominators are the number of remission MRI sessions (167 for MOGAD and 269 for AQP4-NMOSD) instead of the total cohort of patients.

^c^ Included patients receiving subtherapeutic doses of immunosuppression (eg, <5 mg oral prednisolone daily, azathioprine <2.5 mg/kg/d).

### MRI Findings

Within a cohort of 182 patients with MOGAD and 222 with AQP4-NMOSD, a total of 663 MOGAD clinical MRIs were available, 416 performed during an attack (296 sessions) and 247 during remission (167 sessions from 107 patients), and a total of 1048 AQP4-NMOSD MRIs were available, 669 during an attack (470 sessions) and 379 during remission (269 sessions from 136 patients) (Table 1). Follow-up orbital imaging was rare, so data from brain and spinal cord images only were included.

#### Attack-Related New Silent Lesions

In the MOGAD cohort, attack silent lesions were detected in 97 of 296 attack MRI sessions (33%). Of the 265 brain MRIs, 37 (14.0%) found cortical-subcortical silent lesions; 28 (10.6%), brainstem silent lesions; 23 (8.7%), deep white matter silent lesions; 10 (3.8%), deep gray matter silent lesions; 8 (3.0%), cerebellum silent lesions; and 4 (1.5%), optic nerves silent lesions. Of the 151 spinal cord MRIs, 11 (7.3%) found spinal cord silent lesions.

In AQP4-NMOSD, attack silent lesions were found in 88 of 470 (18.7%) attack MRI sessions. Of the 338 brain MRIs, 73 (21.6%) found silent lesions in the deep white matter; 16 (4.7%), in the subcortical white matter; 12 (3.6%), in the deep gray matter; 4 (1.2%), in the brainstem; 4 (1.2%), in the cerebellum; and 1 (0.3%), in the optic nerves. Of the 331 spinal cord MRIs, 3 (0.9%) found spinal cord silent lesions.

#### Remission New Silent Lesions

Median time between last clinical attack and remission MRIs was 9 months (range, 3-25 months) in the MOGAD cohort and 19 months (range, 3-154 months) in the AQP4-NMOSD cohort.

In the MOGAD cohort, new remission lesions were found in 4 of 107 patients (3.7%) in the remission MRI in 5 of 167 remission MRI sessions (3.0%), 2 of which were classified as probable and 3 as definite. Two of the 5 patients (40%) with new remission lesions and 87 of the 162 (53.7%) without them were receiving background immunotherapy (χ^2^_1_ = 0.4, *P* = .54).

One patient had new remission lesions in both the deep white and deep gray matter and subsequently presented with a new remission lesion in the brainstem in a remission MRI; 1 patient had a new remission lesion in deep white matter; 2 patients, in the brainstem; and 1 patient, in the subcortical white matter. With only brain MRIs included in the frequency analysis, brain new remission lesions were found in 4 of 97 patients (4.1%) who had brain new remission MRIs and in 5 of 137 brain remission MRIs (3.6%). With only spinal cord MRIs included in the frequency analysis, spinal cord new remission lesions were found in 0 of 81 patients (0%) who had spinal cord remission MRIs and in 0 of 110 spinal cord remission MRIs (0%). Median time from remission scan to the next relapse in the presence of a new remission lesion (probable and definite) was 2 months (IQR, 1-6 months), and was 2 months for a definite new remission lesion; 4 of 5 patients relapsed at 1, 1, 2, and 6 months, respectively, and the fifth had only 1 month of follow-up with a monophasic course. In the absence of a new remission lesion, median time to relapse was 73 months (IQR, 20-104 months) (hazard ratio, 23.86; 95% CI, 7.51-75.79; Schoenfeld residuals = 0.98; log-rank test *P* < .001) (Figure, A). In the MOGAD patients who relapsed after detection of a new remission lesion, the symptomatic central nervous system areas during the clinical relapses were distinct from the silent lesion locations in 2 of 4 patients.

**Figure.**
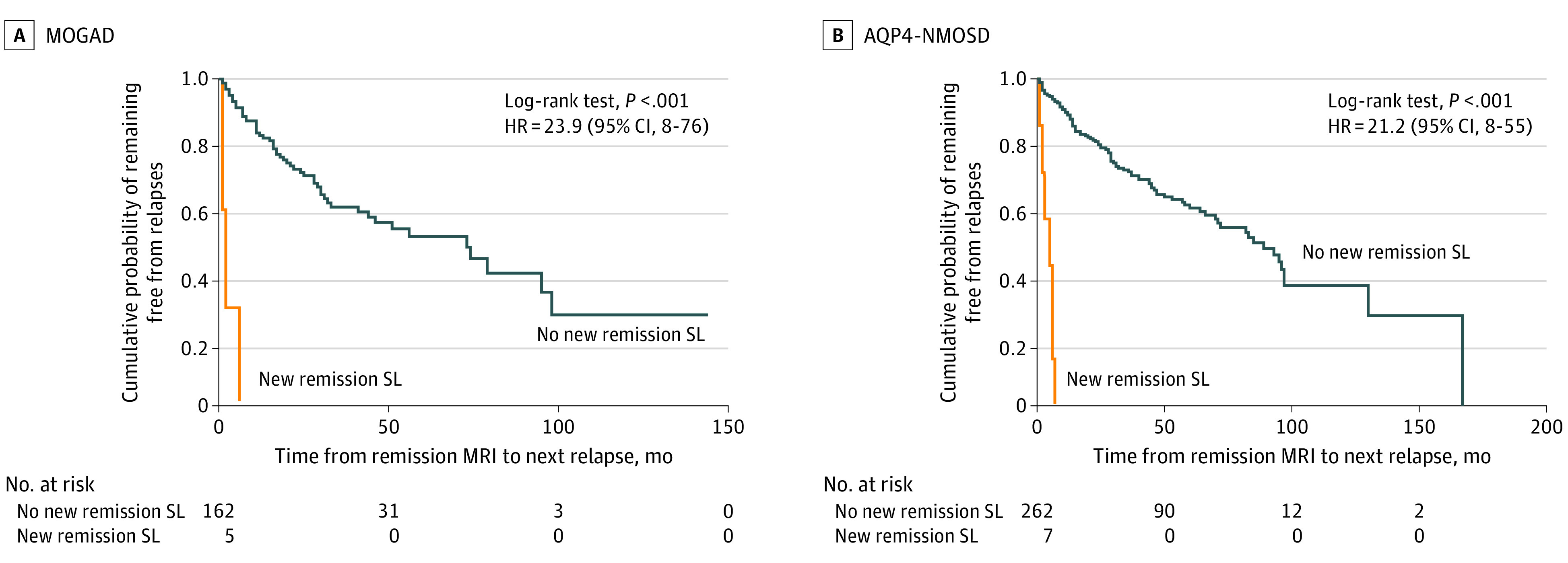
Association Between New Silent T2 Lesions at Remission MRIs and Time to Next Relapse in MOGAD and AQP4-NMOSD Blue line indicates no new remission SL; and orange line, new remission SL. AQP4-NMOSD indicates aquaporin-4 antibody neuromyelitis optica spectrum disorder; HR, hazard ratio; MOGAD, myelin oligodendrocyte glycoprotein antibody disease; MRI, magnetic resonance imaging; and new remission SL, definite and probable new remission silent lesions.

In the AQP4-NMOSD cohort, new remission lesions were found in 6 of 136 patients (4.4%) undergoing remission MRI in 7 of 269 (2.6%) remission MRIs, and 4 were classified as probable and 3 as definite. All AQP4-NMOSD patients were receiving immunosuppressive therapy, although 26 were receiving subtherapeutic doses because they had recently received a diagnosis (Table 1). One patient had 2 new remission lesions in the cervical spinal cord in 2 different remission MRIs (1 was an extension of the previous spinal lesion); 2 patients, in the deep white matter; 2 patients, in the brainstem (1 medulla and 1 midbrain); and 1 patient, in the deep gray matter (thalamus). When only brain MRIs were included in the frequency analysis, brain new remission lesions were found in 5 of 106 patients (4.7%) who had brain remission MRIs and in 5 of 179 brain remission MRIs (2.8%). With only spinal cord MRIs included in the frequency analysis, spinal cord new remission lesions were found in 1 of 116 patients (0.9%) who had spinal cord remission MRIs and in 2 of 200 spinal cord remission MRIs (1%). Median time from remission scan to the next relapse in the presence of a new remission lesion was 5 months (IQR, 2-6 months), and was 3 months for definite new lesions; all 7 patients relapsed at 1, 2, 3, 5, 6, 6, and 7 months, respectively. In the absence of new lesions, median time to relapse was 85 months (IQR, 29-167 months) (hazard ratio, 21.23; 95% CI, 8.05-53.65; Schoenfeld residuals = 0.89; log-rank test *P* < .001) (Figure, B). In the AQP4-NMOSD patients who relapsed after the detection of a new remission lesion, 5 of 7 had symptomatic lesions in the next relapse. There were no systematic differences between remission scans showing new silent lesions compared with those not showing silent lesions that were obvious. Table 2 describes the characteristics of the new silent lesions found on remission MRIs in the MOGAD and AQP4-NMOSD cohorts.

**Table 2.** Characteristics of Remission MRI New Silent Lesions

Type of new SL^a^	Sex	Age at r-MRI scan, y	DMT at r-MRI scan	Time since last attack, mo	Time since reference MRI, mo	New SL CNS location	New SL features
**MOGAD**							
Definite, 1	Female	6	None	15	5	Subcortical in the frontal white matter adjacent to insular cortex	Scattered and ill defined
Definite, 2	Female	41	Pred 7.5 mg once daily	10	6	Splenium of the corpus callosum	Fluffy, ill-defined margins. No gad enhancement.
Definite, 3	Male	14	None	9	5	Left midbrain and cerebral peduncle	Small, ill defined
Probable, 1	Male	51	None	39	39	Anterior pons	Diffuse, ill-defined margins
Probable, 2	Male	13	Pred 25 mg once daily	5	4	Centrum semiovale, corpus callosum, and right internal capsule	Small, ill defined. No gad enhancement.
**AQP4-NMOSD**							
Definite, 1	Female	45	MMF 3 g + pred 20 mg once daily	7	2	Extension of a previous cervical lesion	Swelling and extension
Definite, 2	Male	60	MMF 3 g + pred 25 mg once daily	8	6	Extension of previous brain white matter lesions	Diffuse, confluent lesions. No gad enhancement.
Definite, 3	Female	19	AZA 75 mg twice daily + pred 20 mg once daily	11	6	Area postrema	Faint intensity, normointense on DWI. No gad enhancement.
Probable, 1	Female	45	MMF 3 g + pred 20 mg once daily	5	5	Cervical cord lesion, C1-C4	Longitudinally extensive lesion not observed at the previous attack MRI for thoracic transverse myelitis
Probable, 2	Female	35	Pred 10 mg once daily	66	66	Left parietal white matter and periventricular white matter	Scattered defined deep white matter lesions and a Dawson fingerlike periventricular lesion, DWI hyperintense
Probable, 3	Female	13	RTX 1 g twice, 2 wk apart, every 6 mo) + pred 10 mg adt	5	5	Third ventricle, left thalamus	Small, ill defined
Probable, 4	Female	12	IVIG every 3 wk + pred 20 mg once daily	6	6	Right cerebral peduncle and red nucleus	Small, ill defined

Abbreviations: adt, alternate days; AQP4-NMOSD, aquaporin-4 antibody neuromyelitis optica spectrum disorder; AZA, azathioprine; CNS, central nervous system; definite, new SL found by a remission MRI compared with a reference MRI performed at least 4 weeks after an attack onset; DMT, disease-modifying therapy; DWI, diffusion-weighted imaging; gad, gadolinium contrast; IVIG, intravenous immunoglobulin; MMF, mycophenolate mofetil; MOGAD, myelin oligodendrocyte glycoprotein antibody disease; pred, prednisolone; probable, new SL found by a remission MRI compared with a reference MRI performed at onset or nadir of last clinical attack; r-MRI, remission magnetic resonance imaging (performed at least 3 months from last attack and all had to be completely free of new symptoms); RTX, rituximab; SL, silent lesion.

^a^ Number of patients with definite or probable new remission silent lesions either in MOGAD or in AQP4-NMOSD.

## Discussion

In this retrospective analysis of a large clinical cohort, new silent T2 lesions were found to occur during attacks in both MOGAD and AQP4-NMOSD, but they were more common in MOGAD. However, and in contrast to MS, they were rare in the interval between attacks in both MOGAD and AQP4-NMOSD. This finding is particularly meaningful in the MOGAD cohort, in which approximately half of the patients were not treated at scanning. Conversely, the slightly lower frequency of new remission silent lesions in the AQP4-NMOSD remission MRI cohort compared with the MOGAD cohort may be partly related to the fact that the majority of AQP4-NMOSD patients were receiving immunosuppressive treatment at the time of scanning. The remission new lesions appeared to precede imminent relapses in both cohorts, but their location explained the subsequent relapse symptoms more frequently in AQP4-NMOSD than in MOGAD, demonstrating that many of the “silent” lesions subsequently became symptomatic. Because nonspecific lesions or leukoaraiosis can be misinterpreted as new lesions in MRI of older AQP4-NMOSD patients,^9^ all images were reviewed by NMOSD expert neurologists and neuroradiologists. The new lesions were thought to be inflammatory ones and their relationship with imminent and anatomically relevant attacks would support this.

The presence of brain silent lesions in attack MRIs has been previously noted in smaller cohorts of MOGAD and AQP4-NMOSD patients,^7,8^ and new brain remission lesions were reported in 10 of 74 children with MOGAD,^10^ the majority of which occurred within the first year from onset, but these were not associated with a risk of subsequent relapse. This difference compared with findings in our study might be explained by the inclusion of a higher proportion of relapsing MOGAD patients compared with the solely pediatric cohort.^10^

### Limitations

The main limitation of our study was its retrospective design analyzing remission MRIs performed with different MRI scanners with variable magnetic field intensity and intervals. Moreover, we may have missed some optic nerve silent lesions because they were not scanned regularly. This was not a prospective study, and thus the various intervals between remission MRIs could have affected new remission lesion accumulation, which would be important if lesion accumulation were used as an outcome in a clinical trial (such as in a phase 2 MS clinical treatment study). However, these findings are relevant in actual clinical practice because these 2 diseases are currently managed. The shorter median interval for MOGAD remission MRIs and the greater tendency for lesions to resolve in MOGAD^14^ could have led to an underestimation of new lesions compared with that in AQP4-NMOSD, but the opposite effect might also be true, because more AQP4-NMOSD patients were treated. Only a prospective study performing regular-interval MRI scans with patients not receiving treatments or receiving identical treatments would fairly compare the 2 treatment groups. Additionally, none of these observations remove the conclusion that lesion activity is rare in these antibody diseases within current clinical practice and would not be an appropriate outcome in a clinical trial nor useful in routine monitoring of treatments; nevertheless, new remission lesions, when they do occur, would be a cause for concern.

## Conclusions

The findings of this cohort study suggest that new remission lesions are rare in MOGAD and AQP4-NMOSD and may be associated with imminent relapses. When silent remission lesions occur, they may not remain silent, particularly in AQP4-NMOSD. Given the rarity of new remission silent lesions, the long-term regular monitoring of the lesion load by brain and spinal cord MRI may be not useful either in clinical practice or as a surrogate biomarker of disease activity in clinical trials for MOGAD and AQP4-NMOSD. Nevertheless, in MOGAD compared with MS, consecutive follow-up scans looking for new silent lesions may be a useful diagnostic tool to distinguish MS from MOGAD in patients with low MOG antibody titres, particularly within the first year after disease onset.
